# Adaptation Strategies for Personalized Gait Neuroprosthetics

**DOI:** 10.3389/fnbot.2021.750519

**Published:** 2021-12-16

**Authors:** Anne D. Koelewijn, Musa Audu, Antonio J. del-Ama, Annalisa Colucci, Josep M. Font-Llagunes, Antonio Gogeascoechea, Sandra K. Hnat, Nathan Makowski, Juan C. Moreno, Mark Nandor, Roger Quinn, Marc Reichenbach, Ryan-David Reyes, Massimo Sartori, Surjo Soekadar, Ronald J. Triolo, Mareike Vermehren, Christian Wenger, Utku S. Yavuz, Dietmar Fey, Philipp Beckerle

**Affiliations:** ^1^Biomechanical Data Analysis and Creation (BIOMAC) Group, Machine Learning and Data Analytics Lab, Faculty of Engineering, Friedrich-Alexander-Universität Erlangen-Nürnberg, Erlangen, Germany; ^2^Department of Veterans Affairs, Louis Stokes Clevel and Veterans Affairs Medical Center, Advanced Platform Technology Center, Cleveland, OH, United States; ^3^Department of Biomedical Engineering, Case Western Reserve University, Cleveland, OH, United States; ^4^Applied Mathematics, Materials Science and Technology and Electronic Technology Department, Rey Juan Carlos University, Mostoles, Spain; ^5^Clinical Neurotechnology Lab, Neuroscience Research Center (NWFZ), Department of Psychiatry and Neurosciences, Charité - Universita¨tsmedizin Berlin, Berlin, Germany; ^6^Biomechanical Engineering Lab, Department of Mechanical Engineering and Research Centre for Biomedical Engineering, Universitat Politècnica de Catalunya, Barcelona, Spain; ^7^Institut de Recerca Sant Joan de Déu, Esplugues de Llobregat, Spain; ^8^Department of Biomechanical Engineering, Faculty of Engineering Technology, University of Twente, Enschede, Netherlands; ^9^Department of Physical Medicine and Rehabilitation, MetroHealth Medical Center, Cleveland, OH, United States; ^10^Neural Rehabilitation Group, Department of Translational Neuroscience, Cajal Institute, CSIC, Madrid, Spain; ^11^Department of Mechanical Engineering, Case Western Reserve University, Cleveland, OH, United States; ^12^Chair of Computer Engineering, Brandenburg University of Technology Cottbus-Senftenberg, Cottbus, Germany; ^13^Chair for Computer Architecture, Department of Computer Science, Faculty of Engineering, Friedrich-Alexander-Universität Erlangen-Nürnberg, Erlangen, Germany; ^14^IHP-Leibniz Institut Fuer Innovative Mikroelektronik, Frankfurt (Oder), Germany; ^15^Biomedical Signals and Systems Group, University of Twente, Enschede, Netherlands; ^16^Chair of Autonomous Systems and Mechatronics, Department of Electrical Engineering, Artificial Intelligence in Biomedical Engineering, Faculty of Engineering, Friedrich-Alexander-Universität Erlangen-Nürnberg, Erlangen, Germany

**Keywords:** neuroprosthesis, resistive random access memory, neural interface, personalized devices, perspective, embedded artificial intelligence

## Abstract

Personalization of gait neuroprosthetics is paramount to ensure their efficacy for users, who experience severe limitations in mobility without an assistive device. Our goal is to develop assistive devices that collaborate with and are tailored to their users, while allowing them to use as much of their existing capabilities as possible. Currently, personalization of devices is challenging, and technological advances are required to achieve this goal. Therefore, this paper presents an overview of challenges and research directions regarding an interface with the peripheral nervous system, an interface with the central nervous system, and the requirements of interface computing architectures. The interface should be modular and adaptable, such that it can provide assistance where it is needed. Novel data processing technology should be developed to allow for real-time processing while accounting for signal variations in the human. Personalized biomechanical models and simulation techniques should be developed to predict assisted walking motions and interactions between the user and the device. Furthermore, the advantages of interfacing with both the brain and the spinal cord or the periphery should be further explored. Technological advances of interface computing architecture should focus on learning on the chip to achieve further personalization. Furthermore, energy consumption should be low to allow for longer use of the neuroprosthesis. In-memory processing combined with resistive random access memory is a promising technology for both. This paper discusses the aforementioned aspects to highlight new directions for future research in gait neuroprosthetics.

## 1. Introduction

**Figure 1:**
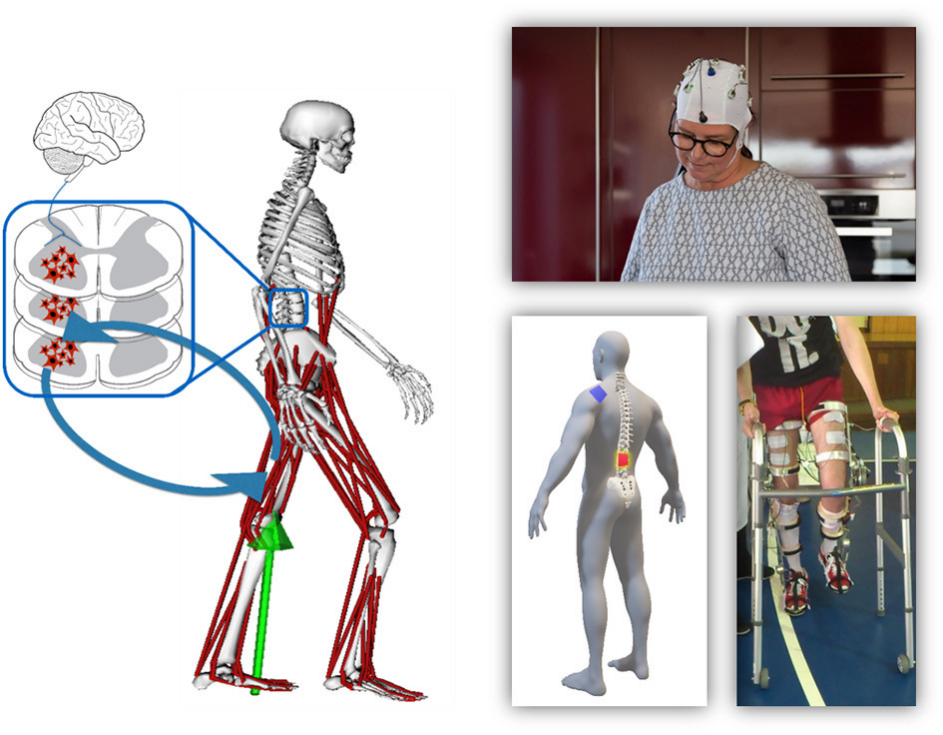
Overview of the different organs involved in gait and their communication structure (left). The right side shows examples of prostheses that interface with these organs, specifically the brain (top), spinal cord (bottom left), or periphery (bottom right).

Gait neuroprostheses aim to restore function in persons with paralysis caused by various injuries, diseases, or dysfunctions in the central or peripheral nervous system, e.g., a stroke, cerebral palsy (CP) or a spinal cord injury (SCI). Interfaces with the brain, spinal cord or the periphery are appropriate (Figure 1), whereas the type and location of the interface is highly dependent on the user's abilities and remaining possibilities for voluntary control. Development of gait neuroprostheses should focus on user-friendliness, and aim to maximize speed and safety, while minimizing fall risk. Furthermore, systems should be portable and easy-to-use to achieve their adoption in real-life environments.

Despite some individual success using interfaces with the brain (e.g., Ajiboye et al. 2017), spinal cord (e.g., Wagner et al. 2018), and periphery (e.g., Nandor et al. 2021), widespread application of neuroprostheses is still limited. One of the main challenges are differences between and within individuals. Between individuals, there is significant heterogeneity in the target population, which requires personalization of stimulation, stimulus timing and intensity, and the possible extent of additional motorized assistance. With “personalization,” we mean that every patient is provided with an individualized neuroprosthesis. This personalization can be done on the hardware and software level. It can be achieved offline through modeling and simulation, or online through adaptation of the stimulation scheme to the patient's current abilities. Personalizing the intervention is currently a time intensive process of trial and error by a human expert. Automation of this process with expert systems that can learn the optimal combinations of muscular and motor activation for a given individual would increase accessibility of gait neuroprostheses (Seel et al., 2016). Over time, due to fatiguing or improved function after rehabilitation, individuals might benefit from different stimulation patterns, which requires stimulation to be adaptive (Del-Ama et al., 2014).

**Figure 2:**
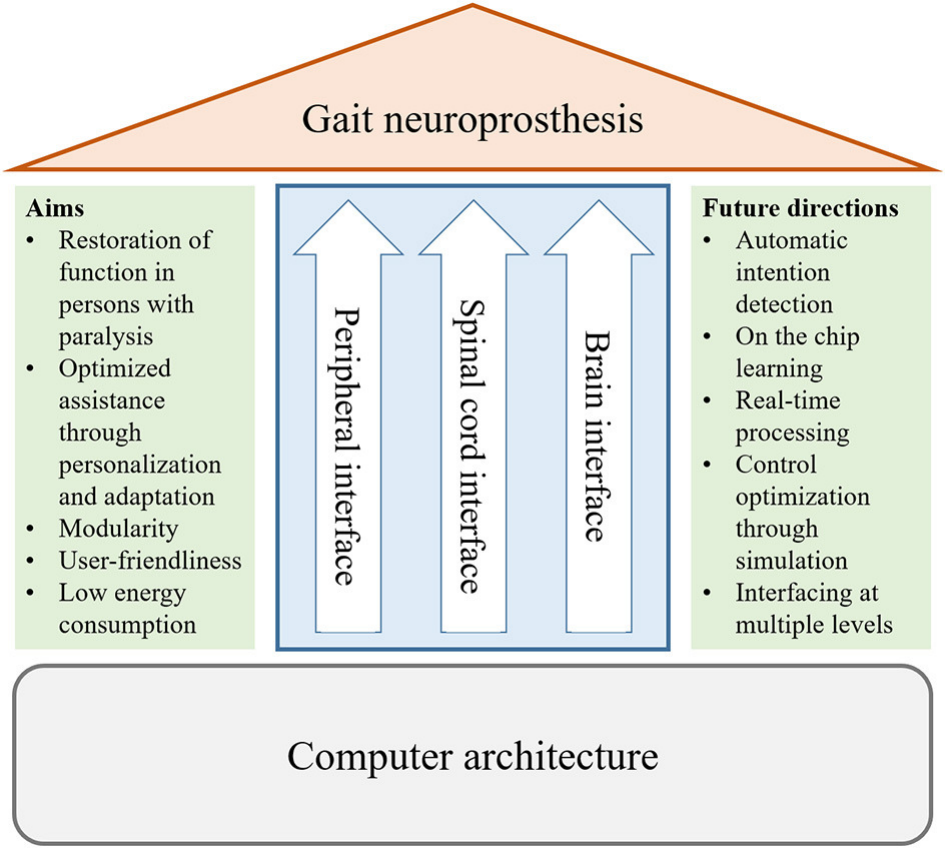
Overview of the topics of this perspective on gait neuroprostheses. We discuss requirements and future directions for interfaces with the periphery and the central nervous system (brain and spinal cord), as well as for the computer architecture.

The development of adaptive interfaces and personalized devices requires an interdisciplinary approach involving clinicians, neuroscientists, engineers, and computer scientists. The effectiveness of gait neuroprostheses can be quantitatively assessed by measuring the gait speed, where desired gait speeds suitable for community use are between 0.8 and 1.2 m/s (Robinett and Vondran, 1988; Lapointe et al., 2001), or by measuring the metabolic energy expenditure while using the device (Asselin et al., 2015; Evans et al., 2015; Miller et al., 2016). Other acceptable metrics could be gait outcomes such as kinematics or symmetry (Hayes et al., 2020). Furthermore, limits for comfortable and tolerable stimulation should be defined for each user. Figure 2 provides an overview of the different aims and future directions that are described in this paper. Movement analyses and simulations are required to understand pathological gait patterns and to tailor assistance, while the design should also be modular and user friendly. Artificial intelligence (AI) would allow control algorithms to be adapted to each individual user, and potentially over the course of rehabilitation. Furthermore, AI has the potential to process data, e.g., electroencephalography and electromyography, accurately in real-time, which is challenging due to the noisiness of these measurements. Adoption of AI in gait neuroprostheses requires development of new software and hardware for efficient on-chip learning, which should also focus on low energy consumption to allow for daily use of the device.

In this perspective paper, the organizers, speakers, and authors of the mini symposium “Adaptation Strategies for Personalized Gait Neuroprosthetics” which was held during the 10^th^ International IEEE/EMBS Conference on Neural Engineering (NER) in May 2021, summarize and extend their contributions and discussions to point out directions for future research. The workshop comprised talks and discussion from experts working on assistive and rehabilitative neuroprostheses and exoskeletons. This perspective covers three topics: interfacing with the periphery, interfacing with the central nervous system, and requirements of interface computing architectures. We discuss and provide a perspective on current research and future directions.

## 2. Interfacing with the Periphery

Interfacing with the peripheral nervous system is a promising method to address specific functional deficits (Wilson et al., 2019). Through user-specific stimulation via surface, percutaneous, or implanted electrodes, muscles can perform a majority of the work for particular tasks. By implementing hybrid actuation approaches combining neural stimulation of affected muscles with robotic assistance, lower extremity function can be restored for patients with gait impairments, who can still drive the main features of the walking cycle by their healthy or preserved muscles. By bringing together neuroprosthetic and wearable robotic approaches, those hybrid concepts combine physiologic benefits by activating the other paralyzed and paretic lower limb muscles through neural stimulation, reducing the demand on external robotic assistance, and augmenting volitional and programmed movements to enhance safety, stability, and endurance.

Neuromechanical simulations of such neuromuscular, biomechanical, and mechanical interactions can be used to optimize the performance of users, hardware design (e.g., reduce size, weight or energy consumption), and controller design (e.g., seamless motor/muscle actuation and appropriate sensory feedback) (Alonso et al., 2012; Crago et al., 2014; García-Vallejo et al., 2016; Uchida et al., 2016; Sreenivasa et al., 2017; Michaud et al., 2019; Sauder et al., 2019). Dynamic simulations of the system composed of the human and the neuroprosthesis can predict the combined human-neuroprosthesis response, allow for device and control customization to maximize walking ability, and improve our understanding of the interaction between human and device for new movement conditions. We advocate to extend computational neuromusculoskeletal models to encompass paralysis-related muscular constraints (Alonso et al., 2012; García-Vallejo et al., 2016) and auxiliary assistive devices (e.g., crutches/walker, orthoses, exoskeletons, etc.) (Febrer-Nafría et al., 2020, 2021) to achieve subject-specific model-based optimization of the device and its control. Then, such simulations might reveal insights into biomechanical effects such as altered recruitment, reduced force production due to atrophy, fatigue effects, or abnormal synergies (Shin et al., 2018). However, creating a personalized neuromusculoskeletal model of a patient is still challenging, since the exact underlying neurological problems of a patient are difficult to extract, and simulations cannot include the variability in muscle responses over time. Experimental validation of simulated clinical outcomes is therefore required (De Groote and Falisse, 2021; Fregly, 2021). Furthermore, even though simulations can provide insight into the required stimulation pattern, the stimulation needs to be adjusted in practice due to the variability in muscle response.

To adapt to the individual user, modular and adjustable hardware and control solutions can be implemented on a joint-need basis, and adapted to different anthropometrics, available muscles and their capabilities, as well as the neurological conditions (Del-Ama et al., 2014; Makowski et al., 2021; Nandor et al., 2021). Control in particular requires an adaptive approach that continuously updates muscle stimulation and electric motor actuation, and potentially prioritizes muscle activity through commanding motors in a trajectory-free ballistic paradigm while monitoring and managing muscle fatigue. To achieve seamless integration of auxiliary assistive devices, control strategies might rely on sensors onboard the human and the machine to measure user-device interaction (Lancini et al., 2016; Ugurlu et al., 2020) and should favor low impedances so the user's muscles can backdrive the device (Foglyano et al., 2016; Beckerle et al., 2017). Wearable kinematic sensing based on inertial measurement units is expected to provide equivalent performance to marker-based approaches. To this end, recent works propose to integrate inertial measurement units into musculoskeletal modeling workflows (Dorschky et al., 2019, 2020; Al Borno et al., 2021; Guidolin et al., 2021). Beyond this, sensory data from the limbs and muscles could be used to improve control and increase muscle perfomance by improved fatigue management compared to current methods based on muscle activity estimation (Alibeji et al., 2017; Mohamad et al., 2017).

## 3. Interfacing with the Central Nervous System

Neural interfacing technologies connected to the CNS, the spinal cord (Wagner et al., 2018) and the brain (Ajiboye et al., 2017) have shown promising results, achieving restoration of control of extremities. While myographic control can provide more reliable and versatile assistance, there is increasing evidence that only stimulation at the CNS level can trigger motor recovery (Soekadar et al., 2015; Cervera et al., 2018). Moreover, when there is no voluntary control of the periphery, an interface to the central nervous system might be the only remaining approach to restore movement or communication (Birbaumer et al., 2014). Most neural interfaces focus on either the brain or the spinal cord, but recently, pioneering studies also investigate combinations, either of the brain and the spinal cord, or the brain and the periphery (Dixon et al., 2016; Shulga et al., 2021).

For the spinal cord, a key goal is to interface with specific motor circuitries responsible for locomotion. Commonly, invasive interfaces are used (Hochberg et al., 2006, 2012; Wagner et al., 2018), which require complex setups to infer neural information of movement intention. Noninvasive approaches, which combine high-density electromyography, blind source separation and neuromechanical modeling, could offer an inexpensive alternative. Although high-density electromyography records spatiotemporal myoelectric activity at the periphery level, it underlies interfering information from spinal neural cells (i.e., alpha motor neurons). Blind source separation (Holobar et al., 2009; Negro et al., 2016) enables separating the interfering activity from neural sources, thereby retrieving the activity of actual motor neuron pools. In turn, the decoded neural firing events can be employed to drive comprehensive neuromusculoskeletal models (Sartori et al., 2016, 2017). Such an interface allows for the activity of actual motor neuron pools to be decoded by separating the interference activity from the neural source. This technique is currently used for characterizing motor neurons during voluntary contractions (Farina and Holobar, 2016) and reflex movements (Yavuz et al., 2015) in healthy and impaired (Holobar et al., 2012) individuals. Moreover, other applications include studying the neuromechanical response to external devices (Farina et al., 2014; Gogeascoechea et al., 2020). This technique can therefore extend current open-loop rehabilitation techniques into closed-loop neuro-modulative approaches. However, its use is mostly limited to isometric contractions or slow dynamic contractions, mainly due to computational challenges related to the assumption that motor units are stationary and real-time implementation of the method. Both model-free AI (e.g., machine and deep learning techniques) (Chen et al., 2020; Clarke et al., 2020) and model-based techniques (e.g., data-driven mechanistic modeling) (Sartori and Sawicki, 2021) are explored to enable real-time implementation, which would allow mechanical and neural adaptations to exoskeleton training and neurostimulation to be predicted.

Besides providing intuitive and seamless assistive control, an important goal at the level of the brain is to promote neuroplastic changes and foster functional connectivity between central motoneurons and inactive and/or silent peripheral motoneurons (Donati et al., 2016). By decoding movement intention and gait characteristics in real time, invasive and non-invasive brain-computer interfaces can directly infer the user's intention to move, optimizing rehabilitation outcomes (Soekadar et al., 2015; Mrachacz-Kersting et al., 2016). Non-invasive brain-computer interfaces can assess large-scale brain oscillatory activity directly, through electroencephalography or magnetoencephalography, or indirectly, by measuring the brain's energy expenditure (Liew et al., 2016; Soekadar et al., 2021). Invasive brain-computer interfaces typically exploit the user's ability to train the electrical activity in their brain, which is recorded by electrocorticography or multielectrode arrays (Hochberg et al., 2012; Ajiboye et al., 2017). The future of individualized brain-computer interfaces interventions relies on advanced algorithms for automated detection of brain states and self-adapting neurofeedback, as well as on hybrid neural interfaces, integrating different biosignals, e.g., electroencephalography, electromyography, or electrooculography, to allow for a more robust and safe control in real-life applications (Witkowski et al., 2014; Soekadar et al., 2016). To enable a broad adoption of brain-computer interfaces in real-life environments for clinics and home use, portable and easy-to-use systems need to be designed, requiring comfortable electroencephalography-headsets that minimize preparation time and allow self-applicability. Furthermore, new machine learning approaches are needed to optimize calibration time without inflating the number of sensors.

Instead of focusing on interfacing solely with either the brain, central nervous system, or periphery, the next generation of gait neuroprostheses for movement rehabilitation may aim to develop an interface on multiple levels. The advantage is that the paired activation of pre- and post-synaptic motoneurons at the level of the spinal cord is crucial for facilitating the re-wiring of functional connections after spinal cord injury (Dixon et al., 2016; Shulga et al., 2021). Motor cortex activity can also be used to control spinal cord stimulation (Capogrosso et al., 2016) instead of external control using a mobile app (Wagner et al., 2018). Adapting the parameters of peripheral stimulation to the ongoing neural activity has also proven to play a key role in fostering neuroplastic reorganization (Mrachacz-Kersting et al., 2016; Bonizzato et al., 2018). Therefore, we envision the implementation of brain-computer interfaces that allow for personalized adaptive modulation of brain activity and alpha motor circuitries.

## 4. Interface with the Embedded Computer Architecture

The main requirements for interface computing architectures of gait neuroprosthetics are that the system is personalizable and adaptable, it should consume as little energy as possible, such that the system can be used optimally for at least a full day, and data processing should happen in real-time. Embedded AI for neuroprosthetics is a promising approach to achieve these requirements, as it has the potential to be real-time, while the computing technology and electronics are attached to the body or even implanted inside the body. The importance of a corresponding computer architecture in neuroprosthesis has been emphasized (Vassanelli and Mahmud, 2016; Ielmini and Wong, 2018), specifically to develop a suitable neuronal architecture to interface with the brain. However, no implementations exist so far, and therefore the further development and prototyping of concepts is of utmost importance (Mikhaylov et al., 2020). To achieve personalization and adaptability, a main challenge is to perform online learning on the embedded AI. In contrast, today's embedded AI only allow inference, while networks are trained offline. This training is computationally intensive and consumes much energy. To achieve low energy consumption and real-time processing for training as well as inference on the chip, the embedded AI should be implemented as low-energy circuits.

An important step to achieve low energy consumption in embedded AI is to use in-memory processing, i.e., operations on data are performed analogously or digitally directly in the memory. Low-energy consumption is inherent for analog processing, since no digital conversion is required, while specialized hardware architectures without a central processing unit can greatly decrease energy consumption since long data paths are avoided. Using non-volatile storage ensures that no information is lost when power is switched off, what can be done when no data processing is required, e.g., when a person wearing a neuroprosthetics stops and does not walk. Accordingly, reloading weights after activation is not required. Many international groups currently study applications of non-volatile memory for neural networks and enhanced computing technologies, e.g., utilizing such memory as synaptic elements for artificial neural networks (Hu et al., 2018; Zhang et al., 2020).

Resistive random access memory (ReRAM) has compared to other non-volatile memory technologies, like e.g., Phase Change Memories, the advantage (Ielmini and Wong, 2018) to further decrease energy consumption, due to a unique combination of multi-level programming and a high density of integration (Milo et al., 2019). Furthermore, ReRAMs are potentially compatible with the most commonly used chip technology, complementary metal–oxide–semiconductor process manufacturing. Multi-level programming means that multiple bits can be stored on one ReRAM device. Therefore, weights and matrix multiplications of a neural network can be stored inside one ReRAM cell, and a neural network can be implemented by saving all network weights directly on the chip (Perez et al., 2020). As a result, external memory access is avoided, which can lower the energy consumption drastically (Knödtel et al., 2020). The high-density integration on the chip allows for the ReRAM cells to be closely attached to digital computation units, which decreases energy consumption by avoiding long data paths on the chip. The use of ReRAM technology yielded a 95% reduction in energy consumption for the classification of bio-signals for atrial fibrillation compared to a traditional approach (Pechmann et al., 2021). Therefore, ReRAM technology is a promising candidate for realization of personalized gait neuroprostheses relying on artificial neural networks in digital (e.g., as ReRAM storage for weights) and analog in-memory processing (e.g., as an in-memory processing element itself).

However, several challenges exist for ReRAM technology to be implemented on a neuroprosthesis. Currently, storage of up to three bits on each ReRAM cell is possible (Milo et al., 2021). However, energy consumption for reading out information increases when more bits are stored on a cell. This trade-off between energy consumption for read out and energy saving from in-memory processing should be further investigated to minimize overall energy consumption. Furthermore, storage elements using ReRAM technology have a larger device-to-device variability than traditional storage elements, which use volatile technology. In particular, this device-to-device variability is a problem for multi-bit storing (Fritscher et al., 2021b), which is desired for embedded AI. Also the variability of switching parameters and energy overheads of analog-to-digital and digital-to-analog conversion is still a challenge for the reliable use of ReRAMs as well as their comparatively low endurance, i.e., the number of allowable switching cycles of the ReRAM cells. This is particularly challgengin for neuroprotheses, which have higher time series data analysis requirements than e.g., a ReRAM based analysator for detecting atrial fibrillation. To achieve this, new research should address special training methods that account for tolerable fluctuations by using learning techniques such as noisy training or dropout layers (Fritscher et al., 2021a).

## 5. Summary and Conclusion

Neuroprostheses can potentially restore function through external activation of the central or peripheral nervous system. We have presented our perspective on current challenges of and future directions in the development of neuroprostheses in stimulation of the peripheral and central nervous systems, and outlined technical approaches to appropriate computer architectures, as also summarized in Figure 2. A paramount challenge is to restore mobility by effectively combining neuroprosthetic and wearable robotic approaches and to align neuroprostheses to the individual user's needs and capabilities.

To this end, predictive simulations of the human-machine system in dynamic tasks appear to be a promising approach to customize design and control. Still, predictive simulations can only provide rough representations of a paralysis and its highly individual constraints, which cannot yet be covered by recent neuromuscular modeling approaches, which, in turn, hampers user-specific control and stimulation. We suggest that future work should focus on improving neuromechanical simulations of user-device interaction based on experimental data, e.g., obtaining muscle parameters on the bench and in real applications, and aim at prioritizing muscle activity over robotic assistance. Combining a deeper understanding of neuromechanical dynamics, particularly muscle-group excitation through the central nervous system, with multimodal sensor networks distributed across the human and the device could foster model-based monitoring and management of muscle fatigue.

For the central nervous system, real-time data processing, ease of use of systems, and combining interfacing at multiple levels are important future directions. Real-time data processing is required to extract useful information from noisy measurements of brain or spinal cord activity in a useful way. AI is a promising approach to enable this real-time data processing, while also allowing for personalization, and limiting the number of sensors required, improving ease of use. Portable and easy-to-use systems should be designed to allow for the adoption of neuroprostheses in real-life. Furthermore, the combination of stimulation at the brain and the spinal cord or the periphery should be further explored, since these combinations were shown to have benefits over stimulation at only one of these levels, and thereby improve rehabilitation outcomes.

Regarding technological advances of the interface to the computing architecture, a main current challenge is to achieve low-energy electronics, real-time data processing, and learning on the chip. Embedded AI has the potential to process data in real-time, and allows for learning and inference on the chip. To achieve low-energy consumption,the embedded AI should use in-memory processing combined with ReRAM technology. To allow the use of ReRAM technology in gait neuroprostheses, we need further research in combining ReRAM technology as multi-bit storage cells with complementary metal–oxide–semiconductor process manufacturing, the commonly-used chip technology. Furthermore, the functionality of the ReRAM cells should be expanded to improve reliability and mitigate the effect its device-to-device variability.

In conclusion, a close cooperation between computer architects, electrical engineers, material scientist, medical experts, and biomechanical experts is required to design appropriate neuroprostheses that are tailored to the user's need, adaptable, easy-to-use, and consume little energy.

## Data Availability Statement

The original contributions presented in the study are included in the article/supplementary material, further inquiries can be directed to the corresponding author.

## Author Contributions

AK coordinated the writing process, the development of the paper structure, and integration of individual contributions. PB and DF supported this for the whole paper. MA, AJd-A, JMF-L, SH, NM, JCM, MN, RQ, R-DR, RJT, and PB mainly contributed to section 2. AC, AG, MS, SS, MV, USY, and AK mainly contributed to section 3. MR, CW, and DF mainly contributed to section 4. All authors contributed to the article and approved the submitted version.

## Funding

AK is funded by a faculty endowment by adidas AG. MA, SKH, NM, MN, RJQ, R-DR, RJT are supported by NSF CPS grant 1739800, VA Merit Reviews A2275-R and 3056, and the NIH (5T32EB004314-15, R01 NS040547-13). MS and AG are funded by the European Research Council (ERC) under the European Union's Horizon 2020 research and innovation programme (Grant agreement No. 803035). AJd-A, JMF-L, and JCM are supported by coordinated grants RTI2018-097290-B-C31/C32/C33 (TAILOR project) funded by MCIN/AEI/10.13039/501100011033 and by “ERDF A way of making Europe”. MR is funded by the Lo3-ML project by the Federal Ministry for Education, Science and Technology (BMBF) (Funding No. 16ES1142K). AC, SS, and MV were supported by the European Research Council (ERC) under the project NGBMI (759370), the Einstein Stiftung Berlin, the ERA-NET NEURON project HYBRIDMIND (BMBF, 01GP2121A and -B) and the BMBF project NEO (13GW0483C).

## Conflict of Interest

The authors declare that the research was conducted in the absence of any commercial or financial relationships that could be construed as a potential conflict of interest.

## Publisher's Note

All claims expressed in this article are solely those of the authors and do not necessarily represent those of their affiliated organizations, or those of the publisher, the editors and the reviewers. Any product that may be evaluated in this article, or claim that may be made by its manufacturer, is not guaranteed or endorsed by the publisher.
